# Unsupervised Cluster Analysis of Walking Activity Data for Healthy Individuals and Individuals with Lower Limb Amputation

**DOI:** 10.3390/s23198164

**Published:** 2023-09-29

**Authors:** Alexander Jamieson, Laura Murray, Vladimir Stankovic, Lina Stankovic, Arjan Buis

**Affiliations:** 1 Department of Biomedical Engineering, University of Strathclyde, Glasgow G1 1XQ, UK; alexander.jamieson@strath.ac.uk (A.J.); laura.murray.100@strath.ac.uk (L.M.); 2 Department of Electronic and Electrical Engineering, University of Strathclyde, Glasgow G1 1XW, UK; vladimir.stankovic@strath.ac.uk (V.S.); lina.stankovic@strath.ac.uk (L.S.)

**Keywords:** activity recognition, clustering, prosthetics, unsupervised learning

## Abstract

This is the first investigation to perform an unsupervised cluster analysis of activities performed by individuals with lower limb amputation (ILLAs) and individuals without gait impairment, in free-living conditions. Eight individuals with no gait impairments and four ILLAs wore a thigh-based accelerometer and walked on an improvised route across a variety of terrains in the vicinity of their homes. Their physical activity data were clustered to extract ‘unique’ groupings in a low-dimension feature space in an unsupervised learning approach, and an algorithm was created to automatically distinguish such activities. After testing three dimensionality reduction methods—namely, principal component analysis (PCA), t-distributed stochastic neighbor embedding (tSNE), and uniform manifold approximation and projection (UMAP)—we selected tSNE due to its performance and stable outputs. Cluster formation of activities via DBSCAN only occurred after the data were reduced to two dimensions via tSNE and contained only samples for a single individual. Additionally, through analysis of the t-SNE plots, appreciable clusters in walking-based activities were only apparent with ground walking and stair ambulation. Through a combination of density-based clustering and analysis of cluster distance and density, a novel algorithm inspired by the t-SNE plots, resulting in three proposed and validated hypotheses, was able to identify cluster formations that arose from ground walking and stair ambulation. Low dimensional clustering of activities has thus been found feasible when analyzing individual sets of data and can currently recognize stair and ground walking ambulation.

## 1. Introduction

Physical activity is universally recommended for the general population and has overwhelming evidence of health benefits [1,2]. This extends also to individuals with lower limb amputation (ILLAs); by maintaining sufficient levels of physical activity, ILLAs will over time see improvements both physically and mentally. From a physical perspective, physical activity can evidently lead to improved heart and lung functionality and reduce the effects of chronic lower back pain [3,4] as well as lead to improved perceptions of the individual’s quality of life, self-esteem, and body image [5,6]. As such, the acquisition of activity monitoring data for the purposes of providing feedback on physical activity for an ILLA population is an invaluable endeavor, which can be achieved through sensing and machine learning, briefly placing the study in a broad context and highlighting its importance.

In particular, this paper explores how unsupervised learning, a machine learning approach that does not use any labeled data, via dimensionality reduction and cluster analysis can be used to support human activity recognition (HAR) that involves an ILLA population. The general objective of an unsupervised learning approach is to present the data in a visualizable format where data points with similarities converge together into clusters. Given the inherent disadvantage of being unable to use the ground truth to influence the classifier behavior, unsupervised approaches tend to underperform compared with supervised approaches when applied to HAR [7,8]. However, since labelling is often impractical and/or expensive, from a clinical perspective, it is worth considering unsupervised approaches to explore whether it is possible to identify unique activity groups within the data features without annotated datasets. In a scenario where clinical researchers want to monitor activity of their patients, they may not have the resources or skills to label data and train a classifier. From that perspective, an unsupervised model would be able to interpret useful data without the overhead of labelling activities, only requiring that the patient wears an activity monitoring device for the time duration of interest.

For a general human population, Ariza-Colpas et al. [9] provided a concise summary of unsupervised approaches to HAR. Unsupervised models that use wearable sensors as primary means of data acquisition are used for the recognition of low-level activities such as standing, sitting, and lying [10,11,12], though a few studies have attempted to distinguish higher-level sporting activities and activities of daily living [13,14,15]. Though numerous sensing and unsupervised approaches have been applied to HAR, there is a clear gap in analysis within an ILLA population, especially in free-living scenarios.

This study aims to close this gap in the literature by proposing the first system for HAR with an ILLA population by clustering relevant physical activities. The key contribution this paper makes is the establishment of clustering scenarios of ILLA data in free-living conditions and the proposal of an unsupervised algorithm, based on t-distributed stochastic neighbor embedding (tSNE)-based dimensionality reduction and density-based spatial clustering (DBSCAN), that can automatically differentiate different walking activities. The contribution of the paper is to explore how unsupervised learning via dimensionality reduction and cluster analysis can be used to support HAR that involves an ILLA population and be able to present the data in a visually interpretable manner. Datasets and Matlab code for this paper are available for public use via the institutional repository [16]. The paper builds on our prior work [17] that proposed supervised classification methods for the recognition of activities carried out by the ILLA population.

The rest of the paper is structured as follows: In Section 2, the methodology of the experiment is explained in terms of how a viable clustering model was obtained and how an unsupervised algorithm was developed to recognize different walking activities. Section 3 details the results of the latter half of the methodology, which is followed by a discussion in Section 4.

## 2. Methodology

### 2.1. Data Collection and Preprocessing

Ethical approval for this study was granted by the University of Strathclyde’s University Ethics Committee prior to conducting the investigation (Ref: UEC20/55). Participants were recruited between September and December 2020 via posters on social media and by contacting individuals who had consented to follow-up participation from previous investigations (see also [17]). Due to a lack of recruitment numbers (primarily caused by the COVID-19 epidemic), subjects were selected based on convenience sampling. All participants had to be at least 18 years of age, be comfortable performing moderate activities, and not be at risk for life-threatening conditions if infected with the coronavirus. The ILLA volunteers were further required to be able to ambulate with a prosthesis without the use of walking aids and not have any comorbidities, which could potentially impact their ability to ambulate for sustained periods of time. As this study was primarily targeted at monitoring the activity of ILLAs, participants were asked not to carry out vigorous activities such as jogging or running.

Recruited participants were instructed to carry out approximately 140 min of walking in the local vicinity of their homes while recording accelerometer data via a thigh-worn device (ActivPAL, PAL Technologies, Glasgow, UK)—see [17], while additionally wearing a chest-mounted camera and recording elevation data via a GPS recording application (Strava, Strava Inc., San Francisco, CA, USA) operating on an iPhone 6 (Apple Inc., Cupertino, CA, USA). The smartphone was provided by the researchers to assist with ground truth annotation of the activities. Activities were annotated using the Visual Object Tagging Tool (VoTT) (Microsoft, Redmond, WA, USA), further using the GPS coordinates from the Strava application to aid with labelling uphill and downhill movement. Note that the ActivPAL accelerometers are used on both legs for amputee participants and only on one leg for able-bodied participants. The obtained labels are used as ground truth for validation. Refer to [17] for more details on the sensing setup, data collection, and cleaning. This investigation was primarily concerned with 5 of the walking activities: level ground walking (referred to as “flat”); uphill movement; downhill movement, and upstairs and downstairs movement. All signal processing and machine learning were handled in a Matlab environment (Matlab 2020b, Mathworks, Natick, MA, USA). The accelerometer data were digitally filtered by a bandpass filter with a preserved frequency range of 0.4 Hz to 3 Hz to remove the gravitational constant and high-frequency muscle artifact movements from the raw signal [18,19]. The data were segmented into chunks of 2 s long (or 40 sample width) windows with no overlap. A list of 243 features (refer to [16]) was constructed for each segment of data and was standardized to have zero mean and a standard deviation of one. Constructed features are in line with those used in the literature for HAR from wearable sensors.

### 2.2. Participant Information

A total of 8 healthy participants with no gait impairment and 4 participants with lower limb amputation were recruited for the study. All participants who consented to participate in the study were able to carry out at least one of their recordings successfully, with zero dropouts. However, 4 individual recordings were discarded from analysis due to inadequate positioning of the recording camera, resulting in poor video quality and making analysis of those recordings infeasible. The primary characteristics of the participants can be found in Table 1 and Table 2. There was a noticeable disparity between healthy and ILLA demographics; while the demographics match in terms of height and weight, there was significant age bias with most of the healthy volunteers being in their mid-20s, while the mean age of the amputees was approximately 50 years and was skewed towards male subjects in both demographics. While this undoubtedly could have had an adverse impact on machine learning testing accuracies, for research purposes it would be useful to determine if training data on a younger healthy population could still result in the detection of activity for an older population with gait impairments. Further, the review in [20] suggests that combining healthy and ILLA populations in supervised HAR studies is commonplace; thus, there is precedence to achieve desirable results. Despite the relatively small sample size of the ILLA subjects, there was some interesting variation in the amputation time with 2 long-term experienced amputees and 2 comparatively inexperienced amputees, and there was a bilateral amputee to provide a comparison point to unilateral amputees. In the following, specific subjects are referred to using a letter-number rule. For example, healthy subject #8 is referred to simply as “H8”, and ILLA subject #2 is referred to as “A2”.

### 2.3. Obtaining Variable Clustering Models

One of the main challenges of working towards achieving a viable cluster model was to consider factors involved in the construction of the low-dimensional cluster model. The main factors to consider are:

- Model Population: Should the data for constructing a cluster model include all participants, split between healthy and ILLA demographics, or create models for each subject individually?
- Dimensionality Reduction: Is it needed, and if so, which dimensionality reduction method is the most appropriate?
- Parameter Tuning: What are suitable hyperparameters for the dimensionality reduction method?
- Label Description and Resolution: Does the detail of the true labels need to be reduced before patterns in the clustering are observed?

The first stage in developing a cluster model is to determine whether the model should include all participants, use separate models for each individual, or split models into ILLA and healthy populations, as well as choosing the appropriate dimensionality reduction method among those commonly used for time series signal clustering, e.g., principal component analysis (PCA), t-distributed stochastic neighbor embedding (tSNE), or uniform manifold approximation and projection (UMAP). For each dimensionality reduction method, the dimensionality is reduced from the original dimensionality (243 features) to 2 dimensions to ensure simple and meaningful visualization and low-complexity implementation. The data points are plotted in a 2D space and are manually labeled post-clustering with their base activity label (flat/level walking, uphill, downhill, upstairs, or downstairs) from ground truth camera observations to indicate the effectiveness of the dimensionality method.

The outcomes of this exercise are shown in Table 3, Table 4 and Table 5, which demonstrate that all dimensionality reduction approaches are only viable when cluster models are created for the individual subject. Note that each color represents one activity. In tSNE and UMAP models, when the activity labels are replaced with the identity of the subject, it becomes evident that these approaches have modeled their cluster formation on the subject, rather than the activity. In contrast, appreciable clustering of activities with combined participant data has been found in the analysis of public HAR datasets [21], implicating that the uncontrolled environment for recording data led to a greater diversification of activities that were unique to each individual. Thus, the chosen approach was to use individual models for cluster analysis.

Table 3, Table 4 and Table 5 further demonstrate that the tSNE and UMAP models were the only models that showed appreciable separability of the activity classes. PCA models completely failed to distinguish any of the 5 classes, and all data points had merged into a singular homogenous shape. When analyzing the explained variance of the PCA components, it was discovered that it took around 70 components to explain 95% of the total systematic variance in the combined dataset of healthy individuals and ILLAs. This finding remains true when PCA is applied to each individual’s dataset separately. It follows that the PCA process is unable to properly distinguish activities with just 2 principal components. This could be because data distribution is far from Gaussian. Due to the instability of the UMAP function for MATLAB 2020b (it would crash the software when run over multiple consecutive executions) [22], it was decided to use tSNE for the remainder of the investigation as it showed similar performance.

Following the initial models generated, though there was appreciable gradient-like separability of activities in the tSNE models, the separation of flat and hill activities was not significant enough to form a cluster. Thus, a reduced level of label resolution was introduced, termed level “0”. In this level of resolution, the flat, uphill, and downhill labels are all consolidated into a single label—“Walk”. The low dimensional clustering results for healthy and ILLA subjects are illustrated in Table 6 and Table 7, respectively. At level 1 of label resolution, for many of the participants, the amount of overlap in flat, downhill, and uphill labels cannot be separated in a practical manner. As a result, the investigation pivoted towards studying clustering activity levels at a much simpler level: distinguishing ground walking (flat, uphill, and downhill) from stairs (up and down).

At resolution level 0, model parameters for the tSNE algorithm were manually adjusted. The tSNE parameters were empirically tuned one parameter at a time, using all 12 subject cluster models and visually comparing them. In general, the choice of parameter had minimal visual improvement on the cluster models over the default configurations, and the models tended to fail to form meaningful clusters when parameters were set to their extreme limits. A configuration of *num. Dimensions = 2, perplexity = 30, exaggeration = 4, and distance = “Standard Euclidean”* was found to be an appropriate solution for all clustering models. Applying PCA prior to tSNE also resulted in poorer cluster models for various levels of principal component preservation, and so it was decided against using PCA in the tSNE process.

### 2.4. Proposed Clustering t-SNE Motivated Algorithm to Distinguish between Walking and Stair Ambulation Clusters

With the grouping of uphill, downhill, and level walking movements into a single label at level 0 resolution, there is a severe class imbalance between walk and stair labels, with walking movement typically encompassing between 90% to 99% of the total data for each individual. Parametric clustering algorithms such as K-Means and Hierarchical clustering, where the number of clusters is predetermined, were deemed ill-fit for cluster recognition, as they typically tended towards forming clusters of equal sizes. Indeed, [23] demonstrated that, regardless of the cluster initialization method, the K-means algorithm performs poorly on unbalanced datasets. It was thus decided to employ density-based spatial clustering (DBSCAN), a nonparametric clustering algorithm, which is more compatible with recognizing clusters with contrasting sizes [24]. Upon initial application of DBSCAN with grid-tuning for optimal parameter setting (found to be, using MATLAB terminology, *epsilon* = 3, *minimum points* = 15), it was evident the DBSCAN algorithm was good at recognizing stair clusters when the stair data was successfully clustered in the tSNE dimensionality reduction process. However, because the upstairs data have not been successfully clustered, the DBSCAN algorithm has no ability to recognize the upstairs sample points as a separate cluster. The immediate disadvantage of the DBSCAN approach is that because the number of clusters cannot be predetermined, the DBSCAN algorithm tends to form additional “false” clusters that simply contain more ground-walking data. To mitigate this problem, a novel algorithm is proposed and described next.

By reducing the classification problem to a binary outcome (a cluster is “walk”, or a cluster is “stairs”), an effective cluster recognition algorithm is developed without the need of using supervised training data. Based on observations of the true labels in the cluster models, the following hypothesis was made:

*I*. *The largest cluster identified by DBSCAN is always a walking cluster.*

**Figure 1:**
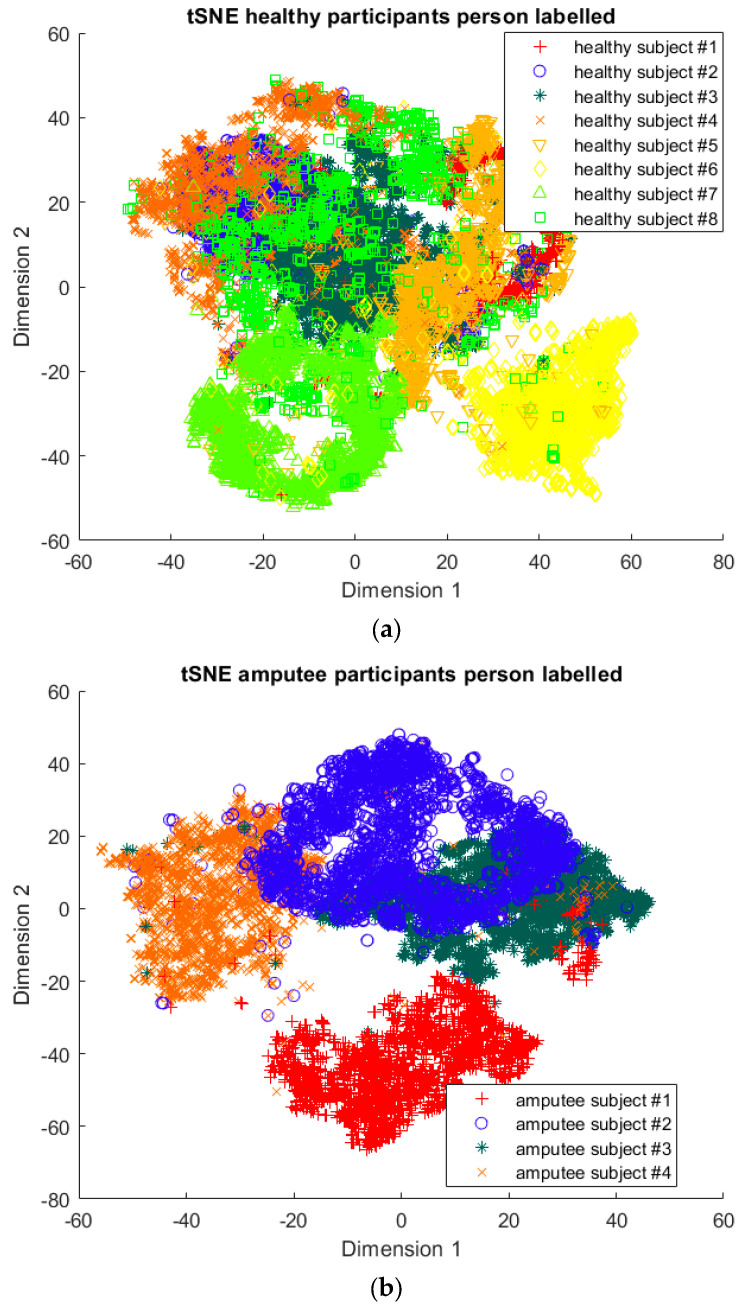
(**a**)-Cluster model of data for all healthy participants, labeled by each individual. (**b**)-Cluster model of data for all ILLA participants, labeled by each individual.

**Figure 2:**
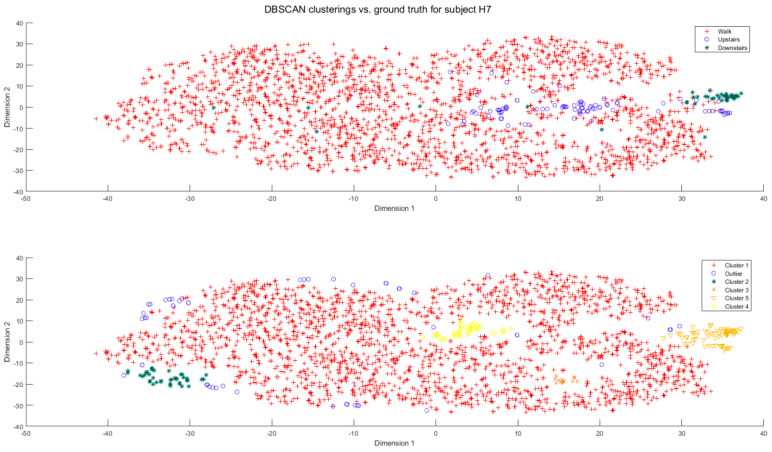
Example of cluster assignments for subject H7 after application of DBSCAN. The top figure represents the ground truth labels, while the bottom represents how clusters are recognized in DBSCAN.

It can be observed in all DBSCAN-related figures in this paper that the largest DBSCAN-identified cluster contains an overwhelming majority of “walk” data points when compared with the ground truth label plots (see Figure 1 and Figure 2). Realistically, there is no practical free-living scenario in which a participant will have more than 20% of their total data based on the stair movement. Thus, the only scenario in which a stair cluster could be the largest DBSCAN cluster is if the volume of input data is too sparse, resulting in many incidental DBSCAN clusters being created. The outlying points detected by the DBSCAN algorithm can also be eliminated from consideration, either as a walking cluster or a stair cluster. If, after applying the DBSCAN algorithm, only one cluster has been identified (excluding errors), the algorithm throws an error.

Usually, there may remain a number of other clusters formed by DBSCAN, thus the objective is then to determine which of these DBSCAN-detected clusters is the “walking” cluster. If one other DBSCAN cluster has been identified (excluding the primary walk cluster and outlier points), this cluster is automatically assigned to the stair cluster by the simple process of elimination. If there are two other (or more) clusters, then the algorithm must identify the DBSCAN cluster with the highest probability of being a stair cluster. The assumption of the algorithm is that only one of these clusters is the stair cluster. All other DBSCAN-detected clusters are assumed to be other “walk” clusters, not recognized as part of the primary walking cluster.

It can be deduced that the DBSCAN-detected cluster *C_i_* that is most likely to be the stair cluster *C_stairs_* is the one that is least likely to be a walking cluster *C_walk_* since:

(1) $$P\left(C_{i}=C_{stairs}\right)=1-P\left(C_{i}=C_{walk}\right)$$

Through further observation of the stair clusters in the tSNE models using ground truth labels, two additional hypotheses were formed:

*II*. *Stair clusters tend to be the clusters with the highest Euclidean distance from the walking cluster.*
*III*. *Stair clusters tend to have more compact clusters than walking clusters.*

To simultaneously validate hypothesis II and develop Equation (1), the distances of clusters can be directly correlated with probabilities; the probability of a DBSCAN-detected cluster being the stair cluster can be calculated as the product of the distance of the cluster’s centroid from the main walking cluster. To calculate this factor, a Gaussian Mixture Model (GMM) with one component is fitted to the main walking cluster, thereby forming a Gaussian distribution around its centroid. The resulting negative log-likelihood of the GMM given the centroid data of each DBSCAN-detected cluster (excluding the already recognized main walking cluster) can then be calculated using posterior probabilities. The negative log-likelihood is a cost function that describes how well a data point or set of data points fit into a machine learning model. The lower the negative log-likelihood, the greater the fit. Thus, clusters with centroids that are located far from the main walking cluster will have high negative log-likelihoods. This will make the data point a poor fit for the walking cluster, thereby being more likely to be the stair cluster.

To validate hypothesis III, the compactness of a DBSCAN-identified cluster is estimated as the median of the pairwise distances between all observations within the cluster. The median was chosen as the summative property to mitigate the effect of any outlier observations in the region.

Using these two hypotheses, an equation for stair cluster probability was composed as the probability of a DBSCAN-detected cluster, *C_i_* being the stair cluster *C_starirs_* as follows:

(2) $$P\left(C_{i}=C_{strairs}\right)=1-\frac{1}{l\left(K_{c_{i}}\in C_{w}\right)\times \varphi_{j\left(C_{i}\right)}}\;(i=1\ldots I)$$

Here, $l\left(K_{c_{i}}\in C_{w}\right)$ represents the negative log-likelihood that a centroid *K* of cluster *C_i_* belongs to the walking cluster *C_w_*. $\varphi_{j(C_{i})}$ represents a ranking factor of the cluster’s compactness. For a clustering model with several potential stair clusters, the cluster with the least compactness is given a value of one. The cluster with the next highest level of compactness is given a value of one plus a scalable reward factor, repeating for all remaining clusters up to the most compact cluster. Once the two main factors have been calculated, the probability of each cluster *C_i_* for all *i* clusters is calculated, and the cluster with the highest probability is assigned as the stair cluster. All other remaining clusters are reassigned as walking clusters.

Given the small number of participants, the number of clusters available for analysis was fairly small. Fortunately, the randomness of the tSNE model-building process can be taken advantage of to help combat the small dataset sizes: by setting the random number generator to a fixed value, the resulting tSNE model is consistently the same when a script is called multiple times, and hence reproducible. The RNG seed was varied from 1 to 5, and the resulting cluster compactness and negative log-likelihoods from each tSNE model were acquired. The statistical analyses of hypotheses II and III are discussed in the Results Section.

### 2.5. Algorithm Summary

In summary, the proposed algorithm steps are as follows. First, the data are collected using ActivPAL and band-pass filtered as described in Section 2.1. The continuous sequence is then segmented into 2 s windows that are used to generate 243 features as in [16]. Each 243-dimensional vector is then projected into 2 dimensions using t-SNE (see Section 2.3). Finally, based on our heuristic analysis of the extracted 2-dimensional data, we apply DBSCAN clustering with the following modifications:

1. If, after applying the DBSCAN algorithm and removing outliners, only one cluster has been identified, the algorithm declares an error.
2. If two clusters are identified, based on hypothesis I, the larger cluster will be labeled as the Walking cluster and the remaining cluster will be the Stair cluster.
3. If multiple clusters are identified, based on hypotheses II and III, Equation (2) is used to estimate the stair cluster probability such that only the cluster with the highest probability is labeled as the Stair cluster. All other clusters are labeled as Walking clusters.

### 2.6. Algorithm Validation

For each subject, the clustering algorithm is evaluated based on two factors:

- Stair cluster purity
- Algorithm-corrected normalized mutual information (NMI).

Stair cluster purity is acquired by reading the labels of the data points of the cluster that has been assigned as the stair cluster, and subsequently calculating the proportion of the number of labels that are either “upstairs” or “downstairs”. Once clusters that do not have the highest stair cluster probability are reassigned to have the same cluster identity as the main walking cluster, the resulting algorithm-corrected tSNE model will only have 2 clusters. This model can then be compared with the ground truth labels and assessed via NMI. The process is run for five unique tSNE model iterations of each subject.

## 3. Results

### 3.1. Validation of Hypotheses

From 5 iterations of tSNE models for each subject, a total of 55 stair clusters and 45 extraneous walking clusters were acquired. From each cluster, the compactness and negative log-likelihood of fitting to the main walking cluster of the respective subject were calculated.

The first stage in statistical analysis was a check for normal distribution, which was achieved through the Shapiro–Wilk test [25]. The *p*-value was found to be less than 0.05 for extraneous walk clusters for both negative log-likelihood and compactness properties (*p* = 9.46 × 10^−4^ and *p* = 0.003, respectively). The same was true for stair clusters for both properties (*p* = 1.7 × 10^−4^ and *p* = 1.22 × 10^−7^); thus, it did not follow normal distribution. As sample distributions were not normal, the Wilcoxon rank-sum test was applied [26]. The significance level was set to 95% (α = 0.05). The null hypothesis for the negative log-likelihood property could be rejected (*p* = 0.023); however, the compactness property could not (*p* = 0.387). This would imply that the distance of the cluster centroid from the main walking cluster is the only significant factor in whether the cluster is a stair cluster or an extraneous walk cluster. It was not desirable to use Equation (2) as univariate, and considering that the compactness *p*-value is significant when a t-test was applied (*p* = 0.026), a compromise was made by making cluster compactness a very weak weighting factor: each cluster in successive order of compactness had an additional rank factor $\left(\varphi_{j(C_{i})}\right)$ of 0.05.

### 3.2. Clustering Algorithm Results

Table 8 presents the purity of stair data counts found within the detected stair clusters. Subject H1 was discarded from analysis due to two of their recordings being carried out with improper wear of the camera, thus having insufficient data for the clustering algorithm. When the stair cluster was correctly recognized, the purity was relatively consistent. There was a clear failure to recognize stair clusters in H3 and A4. The Supplementary Materials Section contains exemplary comparisons of the ground truth activity labels for each subject and comparisons with the algorithm-detected cluster labels. The NMI collected after the algorithm had recognized the stair cluster was compared with the initial NMI obtained through the singular application of DBSCAN in Table 9. While the NMI was still low for most subjects after the application of the algorithm, the algorithm-corrected NMI was found to be greater or equal to the original NMI in all but two of the subjects. To reinforce the positivity of the findings from the algorithm, an additional right-tailed pairwise t-test was carried out to determine, on average across all subjects (excluding H1), whether the algorithm-corrected NMI was greater than the initial NMI after only performing DBSCAN, with the null hypothesis stating that there was no significant difference. The null hypothesis was rejected (*p* = 0.0031), indicating that the proposed algorithm made a systematic improvement in NMI only using DBSCAN.

## 4. Discussion

### 4.1. Clinical Significance

Overall, the experience of attempting to provide a clinically beneficial analysis of free-living activity data of healthy and lower-limb individuals with an unsupervised approach proved to be challenging. Recalling Table 3, Table 4 and Table 5, it was found that when the population model contained multiple participants, the nonlinear dimensionality reduction techniques formed clusters based on the individual rather than the activity. This phenomenon was especially pronounced in the ILLA population model (Figure 1b). Within the ILLA sample group, there was one bilateral amputee and three unilateral transtibial amputees. Accounting just for the three transtibial amputees, there was some diversity in regard to their age and prosthetic experience, both of which can have an impact on gait patterns [27,28]. Given the very small sample size, it is difficult to comment on how the modeling would change if more participants could have been recruited. Furthermore, there was an insufficient number of participants to compare relative findings between healthy individuals and those with ILLA. Regardless, the findings of this investigation imply that an unsupervised approach is only viable by analyzing data from the individual, and not from a general population.

**Figure 3:**
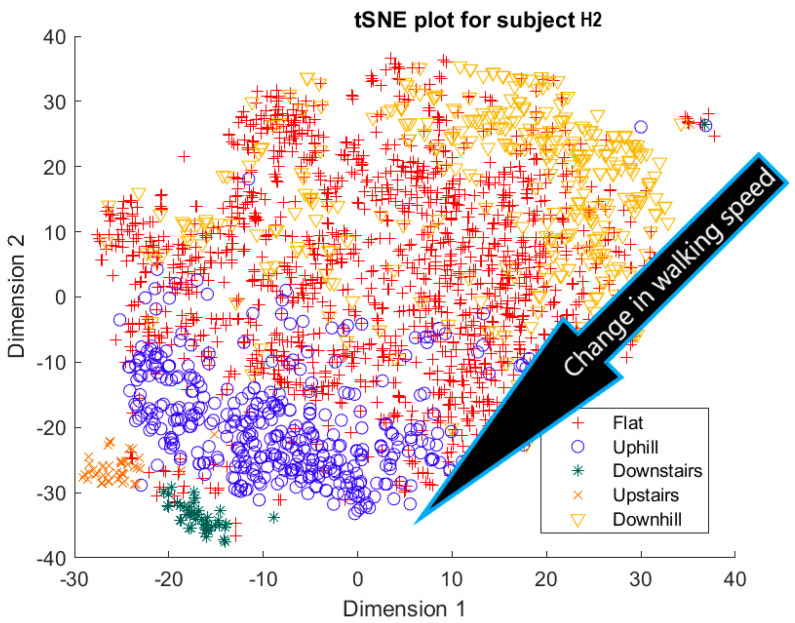
Cluster plot for subject H2 labeled with ground truth activities with the additional highlight of how labels appear to be distributed based on changes in walking speed.

Furthermore, when the true activity labels were investigated, it was found that discernible clustering only existed between stair activity and walking activity, with walking activity encompassing level walking, uphill, and downhill movement. While a few subjects showed an appreciable linear gradient-like distribution of flat, uphill, and downhill movement in the main walking cluster, most subjects did not display this behavior. By observing Figure 3 for subject H2, the downhill labels are distally the furthest away from upstairs and downstairs activities, flat labels are slightly closer, and uphill labels are the closest. These differences may arise because of different natural walking speeds in level walking, hills, and stairs. Unfortunately, it was not possible to validate the walking speed in this study, but gait kinematics literature appears to support this notion. From Sun et al. [29], it was observed that in young adults, walking speed generally tends to increase as the angle of slope decreases. Likewise, it decreases, though to a lesser extent, when traversing uphill. Fujiyama et al. [30] demonstrated that walking speeds on flat surfaces are significantly faster than walking speeds when ascending or descending stairs, even when stairs were being descended quickly, and this was true for both the young adult and elderly population. In ILLA populations, significant differences in preferred walking speed were observed by Rodrigues et al. [31] in transtibial and transfemoral amputees for level walking, uphill, and downhill movement, though interestingly no significant differences in speed were observed in the healthy control group. Finally, Wolf et al. [32] illustrated significant differences in walking speed between ascending ramps and ascending stairs, and descending ramps and descending stairs in ILLAs. These sources indicate that, on a fundamental level, there will be some changes in walking speed between the five main types of ambulation. Therefore, this investigation hypothesizes that successful clustering of stair and hill movement is largely dependable on appreciable changes in walking speed. As the walking speed changes, features relating to the walking speed such as the energy of acceleration and frequency-related features will have significantly different values, which the tSNE algorithm is able to recognize and cluster together in low dimensions. In stair movement, this change in walking speed should be much more appreciable as the subject has to significantly decrease their walking speed to traverse the stairs in a safe manner. Whereas in hill movement, if the angle of the slope is not steep, the subject does not need to make significant alterations to their walking speed, thereby resulting in greater interspersion of the hill and level walking data in the tSNE models. Naturally, this theory will require further testing with validation of the walking speed.

Despite being only able to distinguish two kinds of activities (“walking” and “stairs”), the implications of the investigation could be highly beneficial when applied in the early stages of rehabilitation of an ILLA. A patient, who may have recently acquired a prosthetic fitting for the first time or changed to a new prosthetic component may have trepidations about traversing stairs. Providing a system in which stair traversal activity can be recognized without the need for training or annotating data could be useful for a healthcare professional who wants to evaluate the progress of their clients. Regardless of the findings from the investigation, further experimentation on an ILLA population, particularly those in the early stages of rehabilitation, is warranted to fully comment on the capability of the unsupervised learning approach.

### 4.2. Comparison with Relevant Literature

Studies that are most comparative with this investigation are those that have attempted to differentiate stair and walking activities using only wearable sensors through an unsupervised method for a healthy population. Hunyh [14] was able to distinguish walking and stair movements (upstairs and downstairs separately) with high precision and recall values for each activity. Their sensory setup, however, required a cumbersome and extensive sensory network, with multiple sensors needing to be placed in different locations around the body to acquire good performances. Trablesi et al. [8] used multi-hidden Markov model regression to distinguish walking and ascending stairs with high precision and recall values. Like Hunyh’s research [14], the caveat is that data collection required a multi-sensor array worn across the whole body. Both studies also had significantly smaller numbers of participants than were included in this research, limiting their generalizability to a larger and more diverse population. These studies also appear to have more favorable results by using traditionally supervised-based validation metrics (recognition accuracy) over external clustering validation metrics, such as the NMI used in this study. Given the large class imbalance between stair and walking movement, the existence of outliers within the minority (stair) cluster will proportionately magnify the reduction in mutual information (i.e., the shared labels) between calculated and true cluster identities, thus the cluster quality is higher than the NMI appears to indicate. Finally, both Trablesi et al. [8] and Hunyh [14] studies were carried out in laboratory conditions: this means there was no presence of hill movement (particularly uphill movement), which can potentially muddy the cluster separability between strictly flat walking and stair movement. The literature that has attempted to perform clustering while including ramp activities is very rare, likely due to the difficulty of the task. The only identified study to have attempted to do this with wearable sensors is the work of Kafle and Dou [33], who used EMG sensors and Hierarchical clustering to distinguish level walking, ramps, and stairs. Their highest clustering accuracy achieved was 39.1%, giving a similar performance to this investigation.

### 4.3. Limitations and Future Work

The proposed algorithm can only detect the presence of a stair cluster, and not whether that movement is upstairs or downstairs. In a clinical context, it could be argued that a healthcare professional may only be concerned whether their client is traversing stairs at all, and the direction of movement may be inconsequential. Nonetheless, future iterations of the algorithm would still benefit from recognizing the direction of stair movement. One key factor that strictly limits the generalisability of these findings was that all participants, including ILLAs, used a step-over-step approach to stair ambulation. In a clinical population, ILLAs who have limited prosthetic experience or transfemoral amputation are more likely to use a step-to ambulation approach to improve their stability in gait amputation [34]. Theoretically, this could improve the cluster models as the step-to approach is more different in gait properties than level walking compared with the step-over-step approach but would nonetheless require further testing and validation. Ideally, we would have preferred to carry out a validation study utilizing a stationary motion capture system within a meticulously controlled outdoor setting. Regrettably, this became unfeasible due to the constraints imposed by the pandemic situation.

## 5. Conclusions

This investigation has covered the exploration of an unsupervised clustering approach to distinguish walking activities for healthy and ILLA populations in free-living conditions. After studying the clustering behavior and compromising on label resolution, a novel algorithm based on combining DBSCAN cluster models with a probability equation was created to identify the presence of stair movement, which could be clinically beneficial for monitoring the progress of ILLAs in the early stages of rehabilitation. The significance of the cluster analysis findings lies in its ability to discern the surface on which a prosthetic user is walking solely through the analysis of activity data in real-world scenarios, eliminating the need for costly and intricate motion analysis equipment. Accurate monitoring and evaluation of the real-world activity of individuals with lower limb amputation can be complex. Prosthetic users can often over- or under-report their true activity. The ability to evaluate their activity and the environmental barriers they encounter is a critical component of making an accurate prescription.

Future work should provide validation of the subject’s walking speed as a potential confounding factor on cluster model quality and include volunteers where the step-to approach is their preferred method for stair ambulation. The final aim is to create an initial “health” system for prosthetics that can prevent significant complications for both the user and the healthcare system.

## Figures and Tables

**Table 1:** Characteristics of healthy participants with no gait impairment.

Subject	Height (m)	Weight (kg)	Age (Years)	Gender
Healthy Subject #1	1.80	84	24	Male
Healthy Subject #2	1.65	63	51	Female
Healthy Subject #3	1.62	65	18	Female
Healthy Subject #4	1.97	99	25	Male
Healthy Subject #5	1.92	102	25	Male
Healthy Subject #6	1.83	89	24	Male
Healthy Subject #7	1.84	88	25	Male
Healthy Subject #8	1.78	98	25	Male

**Table 2:** Characteristics of individuals with lower limb amputation.

Subject	Height (m)	Weight (kg)	Age (Years)	Gender	Type of Amputation
Amputee Subject #1	1.79	95	55	Male	Unilateral transtibial
Amputee Subject #2	1.70	86	57	Male	Unilateral transtibial
Amputee Subject #3	1.72	110	40	Male	Unilateral transtibial
Amputee Subject #4	1.52	61	48	Female	Bilateral transtibial

**Table 3:** PCA−generated cluster models of activity data for each population model.

Data Population	PCA Model
All subjects	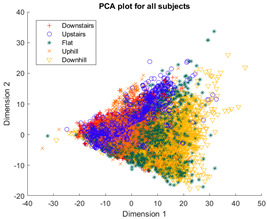
Healthy subjects	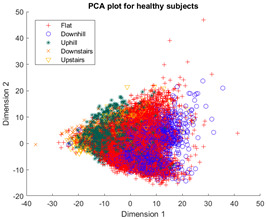
ILLA subjects	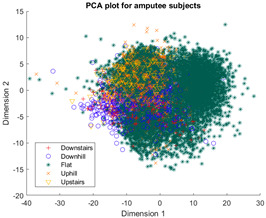
Individual subjects (H1)	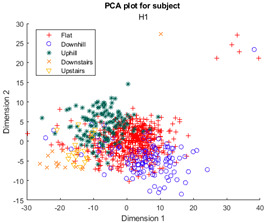

**Table 4:** tSNE−generated cluster models of activity data for each population model.

Data Population	tSNE Model
All subjects	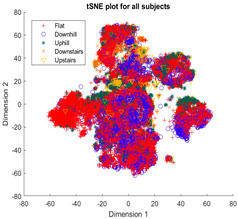
Healthy subjects	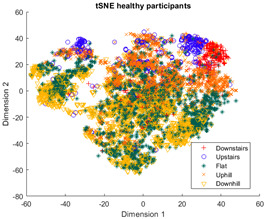
ILLA subjects	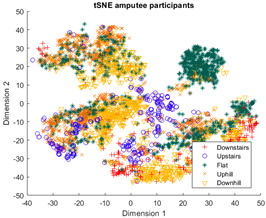
Individual subjects (H1)	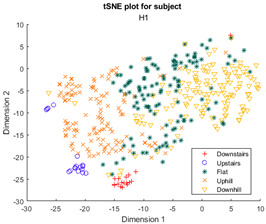

**Table 5:** UMAP−generated cluster models of activity data for each population model.

Data Population	UMAP Model
All subjects	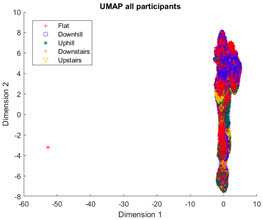
Healthy subjects	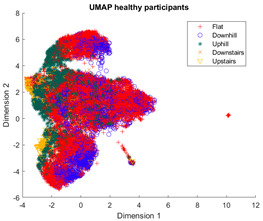
ILLA subjects	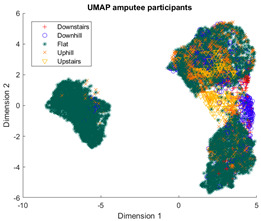
Individual subjects (H1)	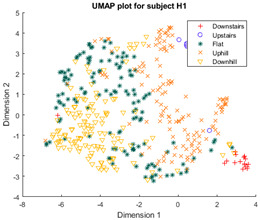

**Table 6:** Clustering at different levels of label resolutions for one healthy subject (H2).

Resolution Level	Healthy Subject Cluster Model
0 (Walking and stairs)	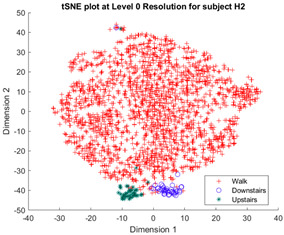
1 (Flat walking, hills, and stairs)	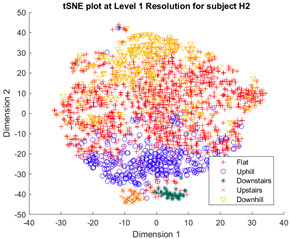

**Table 7:** Clustering at different levels of label resolutions for one ILLA subject (A2).

Resolution Level	ILLA Subject Cluster Model
0 (Walking and stairs)	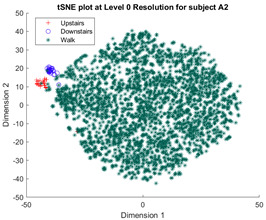
1 (Flat walking, hills, and stairs)	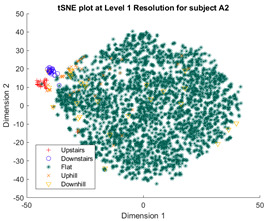

**Table 8:** Mean stair cluster purity percentage for each subject and each model.

Subject	H2	H3	H4	H5	H6	H7	H8	A1	A2	A3	A4
Mean	38.95	0	85.11	65.99	79.26	72.38	26.45	70.12	86.82	71.25	9.41
Std.	38.15	0	0	33.1	39.66	7.96	33.84	0.78	5.59	16.36	18.82

**Table 9:** NMI of cluster model after application of an algorithm compared with the initial application of only DBSCAN.

Subject	H2	H3	H4	H5	H6	H7	H8	A1	A2	A3	A4
Initial NMI	0.279	0.01	0.266	0.419	0.377	0.226	0.149	0.538	0.595	0.168	0.023
Std.	0.176	0.002	0.022	0.091	0.125	0.026	0.046	0.07	0.057	0.038	0.044
Final NMI	0.374	0.01	0.367	0.433	0.377	0.216	0.164	0.538	0.608	0.155	0.027
Std.	0.133	0.001	0.048	0.086	0.125	0.017	0.078	0.046	0.064	0.052	0.052

## Data Availability

The accelerometer data presented in this study are openly available in Alexander Jamieson’s PURE account, https://pureportal.strath.ac.uk/en/datasets/data-for-construction-of-a-clinical-activity-monitoring-framework (accessed on 20 September 2023) see Jamieson [16]. The video files of the original recordings are not included in this dataset to protect the privacy of the participants.

## Supplementary Materials

The following supporting information can be downloaded at: https://www.mdpi.com/article/10.3390/s23198164/s1.
