# Supplementary material for: Unsupervised Cluster Analysis of Walking Activity Data for Healthy Individuals and Individuals with Lower Limb Amputation
Source: Sensors (Basel). 2023 Sep 29;23(19):8164. doi: 10.3390/s23198164 (PMC10575014; doi:10.3390/s23198164)
Supplement: Supplementary file 1 [file sensors-23-08164-s001.zip › sensors-2456503-supplementary.pdf]

This Supplementary Material demonstrates exemplary results of how activity labels were clustered by the tSNE algorithm (the ground truth), and the resulting cluster model developed by the proposed algorithm. This is applied for all subjects barring subject H1. Note that these figures are not an accurate representation of the numerical results obtained in this investigation (the obtained NMIs from Table 9) and are simply for illustration purposes. As a disclaimer, the in-figure title of each figure has been edited to remove the original codename of the subject and is replaced with the codenaming procedure used in the main article.

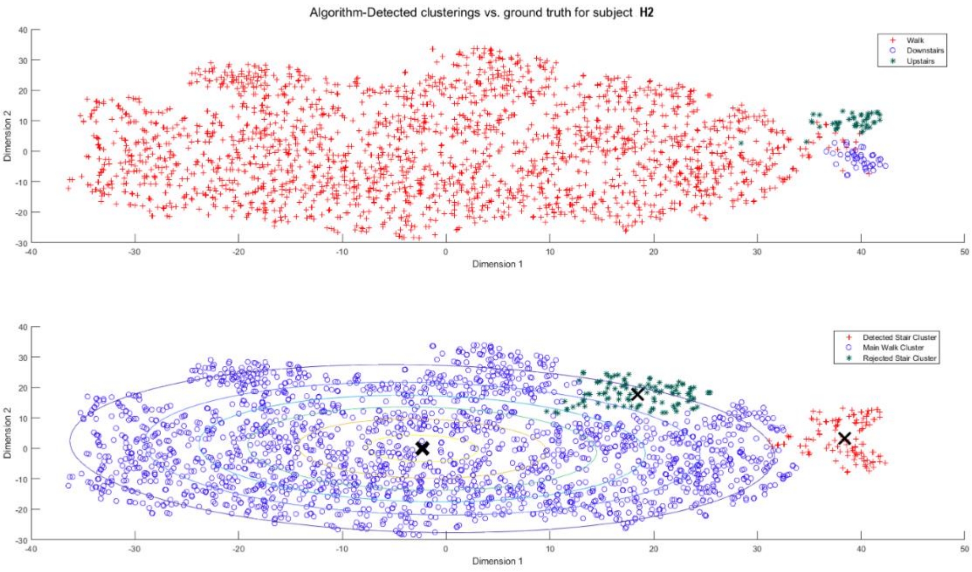

Figure S1. The top figure represents the ground truth scenario of the subject H2, where each data point is represented by its ground truth activity label. The bottom figure represents the same datapoints as the top figure but is changed to reflect how the algorithm devised in the article identifies the main activity clusters. Only one cluster is recognized as the stair cluster, while all others represent level walking or walking on slopes. The coloured rings visualize the probability density function of the largest walk cluster, emanating from the centroid of the largest walk cluster, identified by the black cross. The other black crosses represent the centroids of other clusters.

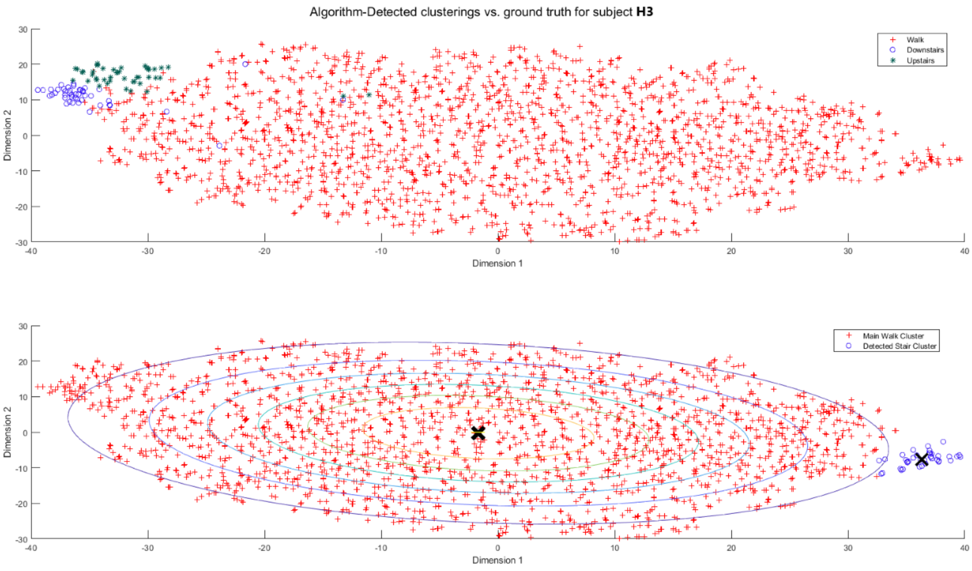

Figure S2. The top figure represents the ground truth scenario of the subject H3, where each data point is represented by its ground truth activity label. The bottom figure represents the same datapoints as the top figure but is changed to reflect how the algorithm devised in the article identifies the main activity clusters.

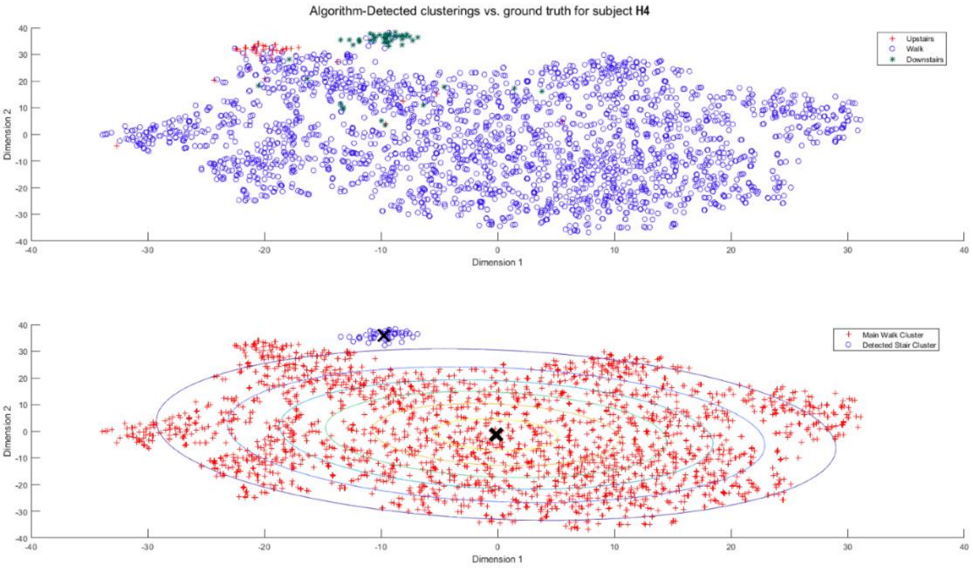

Figure S3. The top figure represents the ground truth scenario of the subject H4, where each data point is represented by its ground truth activity label. The bottom figure represents the same datapoints as the top figure but is changed to reflect how the algorithm devised in the article identifies the main activity clusters.

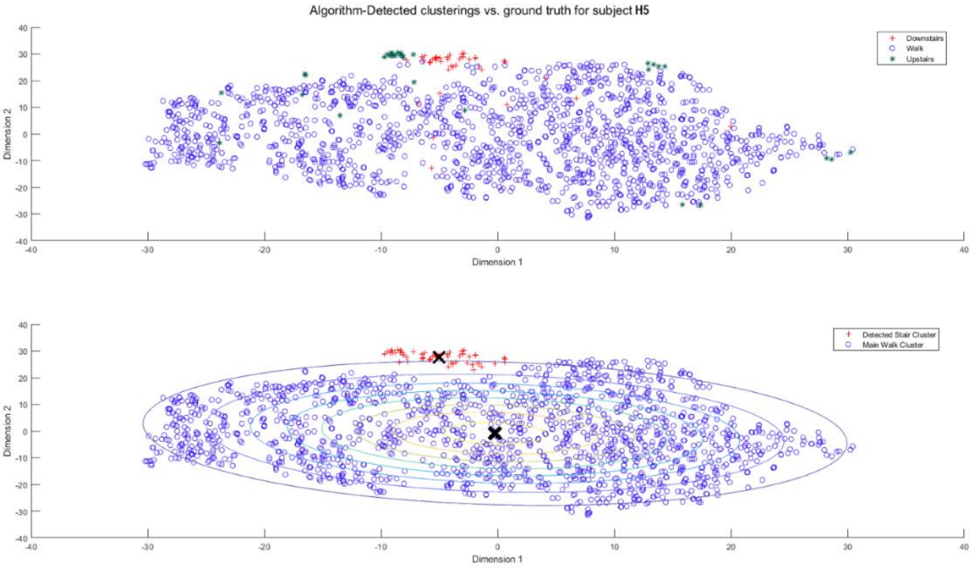

Figure S4. The top figure represents the ground truth scenario of the subject H5, where each data point is represented by its ground truth activity label. The bottom figure represents the same datapoints as the top figure but is changed to reflect how the algorithm devised in the article identifies the main activity clusters.

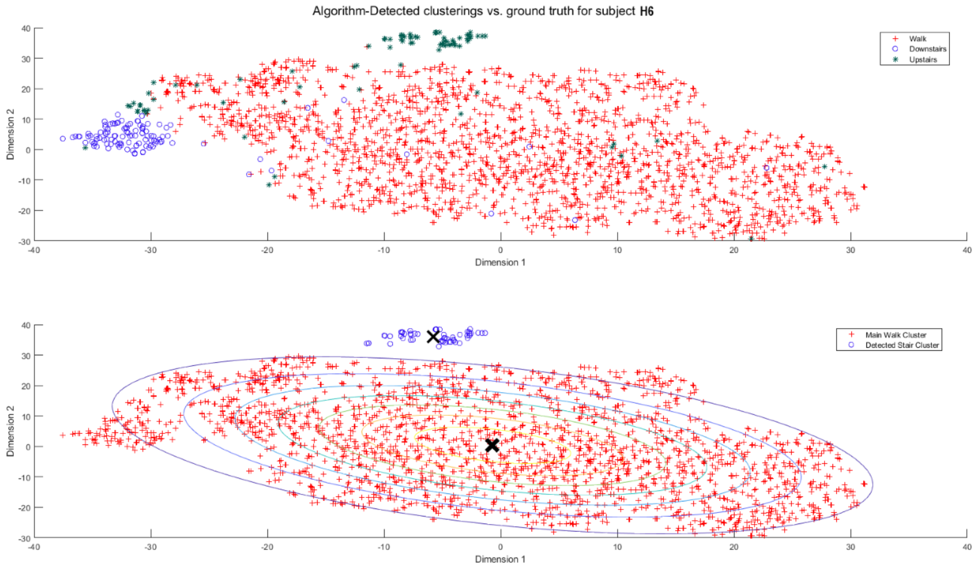

Figure S5. The top figure represents the ground truth scenario of the subject H6, where each data point is represented by its ground truth activity label. The bottom figure represents the same datapoints as the top figure but is changed to reflect how the algorithm devised in the article identifies the main activity clusters.

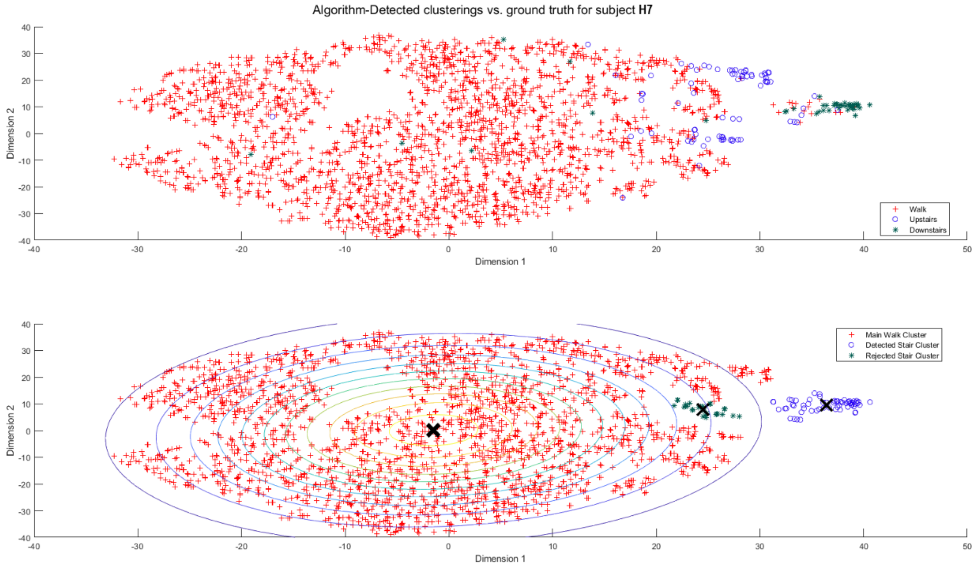

Figure S6. The top figure represents the ground truth scenario of the subject H7, where each data point is represented by its ground truth activity label. The bottom figure represents the same datapoints as the top figure but is changed to reflect how the algorithm devised in the article identifies the main activity clusters.

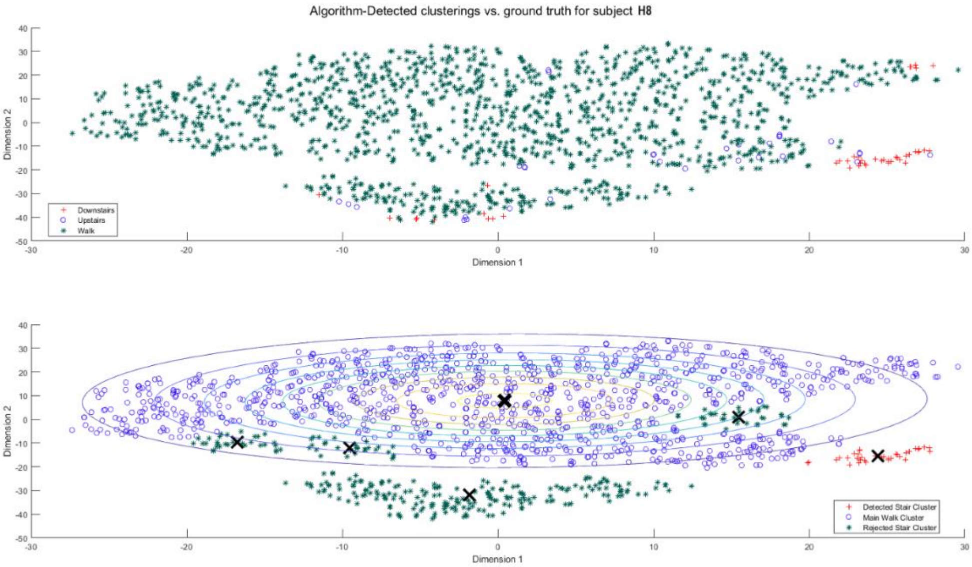

Figure S7. The top figure represents the ground truth scenario of the subject H8, where each data point is represented by its ground truth activity label. The bottom figure represents the same datapoints as the top figure but is changed to reflect how the algorithm devised in the article identifies the main activity clusters.

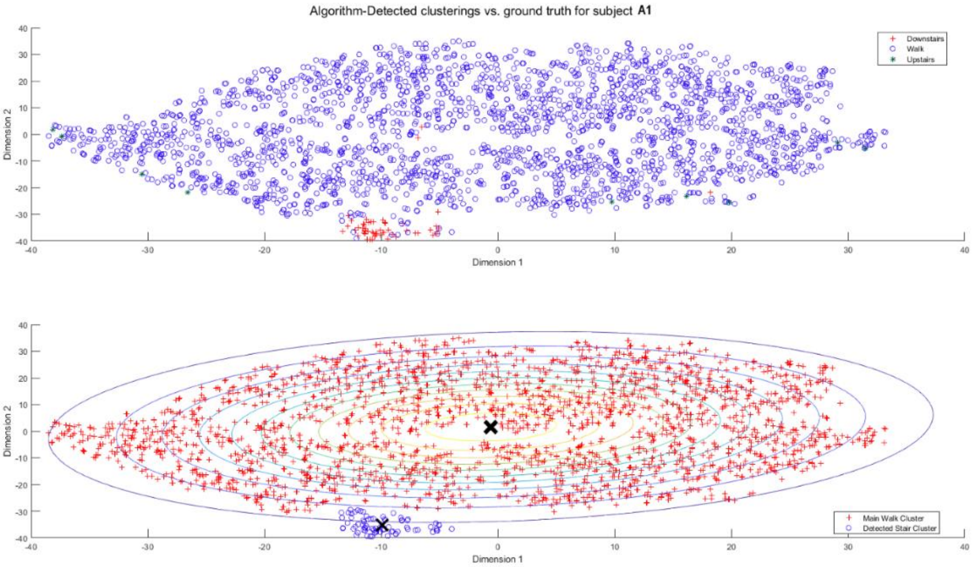

Figure S8. The top figure represents the ground truth scenario of the subject A1, where each data point is represented by its ground truth activity label. The bottom figure represents the same datapoints as the top figure but is changed to reflect how the algorithm devised in the article identifies the main activity clusters.

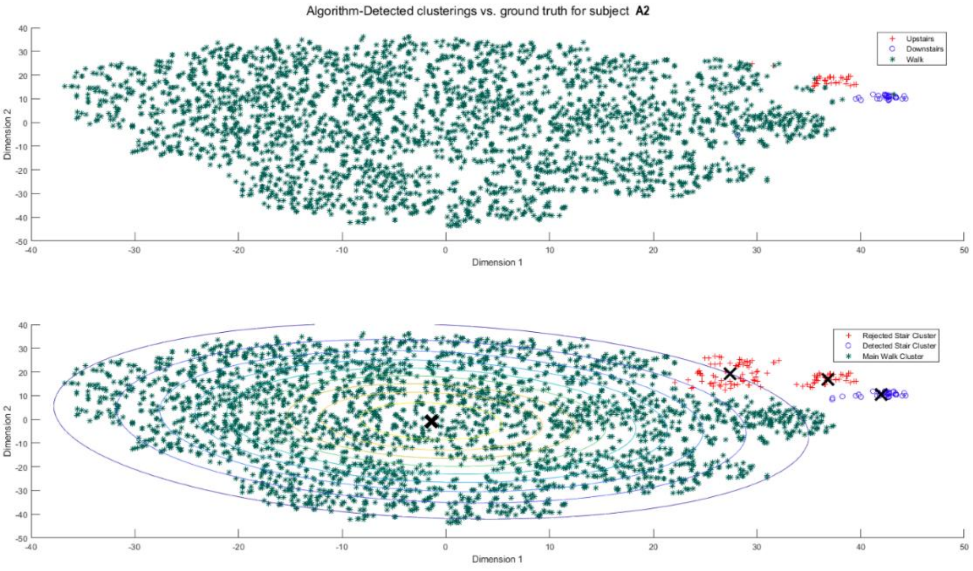

Figure S9. The top figure represents the ground truth scenario of the subject A2, where each data point is represented by its ground truth activity label. The bottom figure represents the same datapoints as the top figure but is changed to reflect how the algorithm devised in the article identifies the main activity clusters.

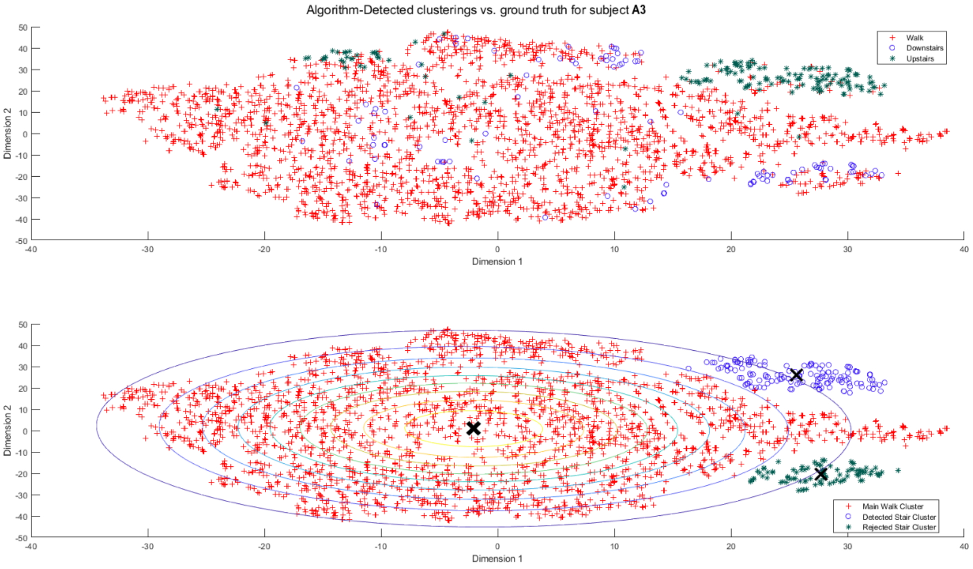

Figure S10. The top figure represents the ground truth scenario of the subject A3, where each data point is represented by its ground truth activity label. The bottom figure represents the same datapoints as the top figure but is changed to reflect how the algorithm devised in the article identifies the main activity clusters.

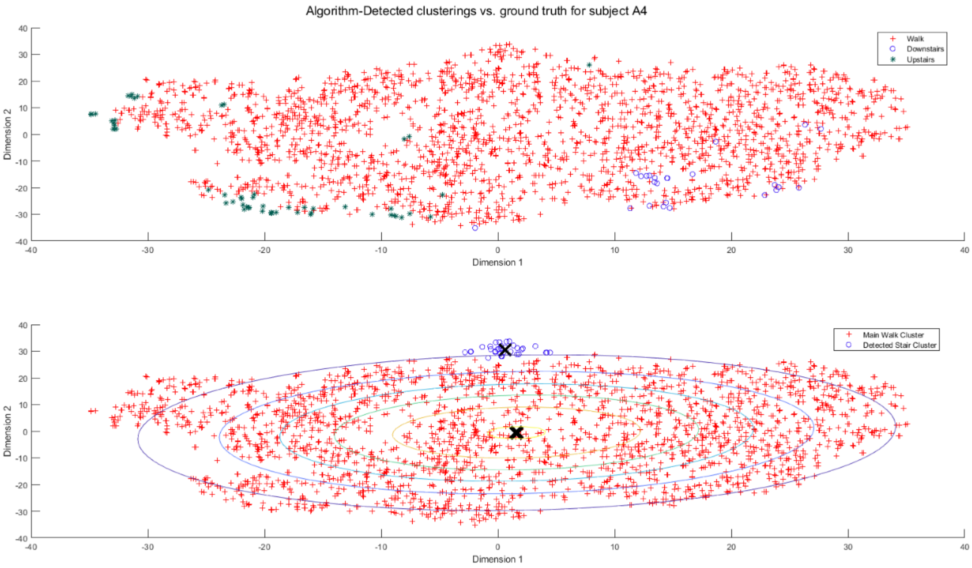

Figure S11. The top figure represents the ground truth scenario of the subject A4, where each data point is represented by its ground truth activity label. The bottom figure represents the same datapoints as the top figure but is changed to reflect how the algorithm devised in the article identifies the main activity clusters.
